# The nocebo effect as a source of bias in the assessment of treatment effects

**DOI:** 10.12688/f1000research.17611.2

**Published:** 2019-03-11

**Authors:** Karolina Wartolowska

**Affiliations:** 1 Nuffield Department of Primary Care Health Sciences, University of Oxford, Oxford, OX2 6GG, UK

**Keywords:** Review (article), Nocebo Effect, Placebo Group, Adverse Events in Clinical Trials, Randomised Clinical Trial (RCT)

## Abstract

The term nocebo effect refers to the harmful outcomes that result from people’s negative beliefs, anticipations, or experiences related to the treatment rather than the pharmacological properties of the treatment. These outcomes may include a worsening of symptoms, a lack of expected improvement, or adverse events, and they may occur after the active treatment and the placebo that is supposed to imitate it.  The nocebo effect is always unwanted and may distort estimates of treatment effectiveness and safety; moreover, it may cause discontinuation of therapy or withdrawal from a trial.

The nocebo effect may be unintentionally evoked by the explanations given by healthcare professionals during a clinical consultation or consent procedures, or by information from other patients, the media, or the Internet. Moreover, it may be a consequence of previous bad experiences with the treatment, through learning and conditioning, and the conditioning may happen without patients’ conscious awareness. In trial settings, a study design, for example lack of blinding, may introduce bias from the nocebo effect.

Unlike the placebo effect, which is usually taken into consideration while interpreting treatment outcomes and controlled for in clinical trials, the nocebo effect is under-recognised by clinical researchers and clinicians. This is worrying, because the nocebo phenomenon is common and may have potentially negative consequences for the results of clinical treatment and trials. It is therefore important that doctors and medical researchers consider any potential nocebo effect while assessing the treatment effect and try to minimise it through careful choice and phrasing of treatment-related information given to patients.

## Introduction

Nocebo is often described as placebo’s evil twin, and is rarely discussed on its own. This phenomenon is under-recognised in clinical practice and clinical trials, and many patients and healthcare professionals admit that they are not aware of its existence ( Berthelot *et al.*, 2001).

The nocebo effect is defined as the adverse effects of an intervention that are not related to its pharmacological or physiological effects. In research settings, it refers to the negative effects of a placebo. In clinical or trial settings, the term is used to describe the negative effects produced by placebos or either harm or lack of efficacy of the active intervention ( Benedetti *et al.*, 2007; Hahn, 1997; Häuser *et al.*, 2012). In this paper I will not distinguish between a nocebo effect and the nocebo response and I shall refer to all the clinical/psychological or physiological changes in a patient or a group of patients that are not related to the pharmacological or physiological effect of the treatment as the nocebo effect.

The nocebo effect is mediated through negative emotions such as stress, fear, and anxiety ( Aslaksen & Lyby, 2015; Benedetti *et al.*, 2006; Bingel *et al.*, 2011) At the physiological level, it is associated with activation of the hypothalamic-pituitary-adrenal (HPA) axis, which controls reactions to stress, and with higher concentrations of the “stress hormone”, cortisol ( Benedetti *et al.*, 2006; Johansen *et al.*, 2003). Both the nocebo effect and HPA hyperactivity are reduced by anxiolytic drugs ( Benedetti *et al.*, 2006). Anticipatory anxiety facilitates pain transmission, at least partly through cholecystokinin receptors ( Benedetti *et al.*, 1995; Lovick, 2008) and causes nocebo hyperalgesia ( Bingel *et al.*, 2011; Keltner *et al.*, 2006). The nocebo effect is also associated with reduced activation of dopaminergic and opioidergic systems ( Scott *et al.*, 2008; Svedman *et al.*, 2005).

## Nocebo effect in clinical practice

The consequences of the nocebo effect in clinical practice are always undesirable. It may make therapeutic interventions more painful, reduce responses to treatment, worsen symptoms or lead to adverse events, in turn causing non-adherence or discontinuation of treatment ( Blasini *et al.*, 2017). For example, an injection of an epidural analgesic can be made more painful when patients are warned that it would feel like a bee sting rather than told only that it would create a numbing sensation ( Varelmann *et al.*, 2010). Similarly, using the word “pain” rather than “a cool sensation” in a description of a procedure may make this procedure painful ( Lang *et al.*, 2005). Also, the efficacy of pharmacologically active substances was greatly reduced when they were given with contradictory information, for example bronchoconstrictors as reducing asthma and bronchodilators as provoking it ( Luparello *et al.*, 1970). Information that injection of a powerful opioidergic analgesic was started or stopped increased or decreased its analgesic effects, despite continued delivery of the drug ( Bingel *et al.*, 2011). In another study, pain ratings after the suggestion of hyperalgesia were higher than after the suggestion of analgesia, regardless of whether they were accompanied by an application of an analgesic cream or a placebo ( Aslaksen *et al.*, 2015).

The nocebo effect may also be responsible for worsening of symptoms or for new symptoms, which are often recorded as adverse effects related to the medication. For example, nebulised saline evoked asthma attacks in patients with asthma if it was given with information that contained an irritant, while the same saline relieved the symptoms if it was presented as an active treatment ( Luparello *et al.*, 1968). This is particularly important in the context of clinical trials, as a patient may be asked to record any unusual symptoms and the doctor, who may not know the patient’s long-term clinical history, may be more likely to interpret any new symptom as an adverse effect of the medication. Adverse events caused by the nocebo effect are discussed in more detail in the section on nocebo in clinical trials.

In clinical settings, the nocebo effect can be easily evoked through verbal suggestion ( Benedetti *et al.*, 2007). These negative effects are usually created unintentionally, by the description of the treatment effects and adverse effects during a clinical consultation or during a consent process ( Benedetti *et al.*, 2007; Tobert & Newman, 2016; Vase *et al.*, 2011) The information does not have to be given directly, but may be written on a drug leaflet or patient information letter. The negative verbal suggestions may also come from sources without medical authority, such as other patients ( Colloca *et al.*, 2004), friends and family, or the media and the Internet ( Crichton & Petrie, 2015). For example, patients in countries where they are more likely to find websites about the adverse effects of statins are more likely to demonstrate statin intolerance ( Khan *et al.*, 2018). Such negative information may cause negative emotions and negative expectations about the outcomes of treatment ( Häuser *et al.*, 2012)

The nocebo effect may also be created non-verbally. It may result from observing doctors’ body language ( Häuser *et al.*, 2012) or by observing the symptoms, adverse effects, and behaviour of other patients undergoing the treatment ( Colloca & Benedetti, 2009; Hahn, 1997; Swider & Babel, 2013). Having one’s treatment stopped may also cause the nocebo effect ( Bingel *et al.*, 2011; Colloca *et al.*, 2004).

It may also result from one’s bad experiences, through learning and conditioning. The nocebo effect may be caused by dissatisfaction with past treatment, for example due to lack of treatment efficacy or adverse effects ( Kessner *et al.*, 2013). This creates explicit negative expectations and attitudes that shape future responses ( Faasse & Petrie, 2013). For example, patients may assume that if the treatment didn’t work in the past, it won’t work this time, or they may believe that they do not tolerate the treatment ( Tobert & Newman, 2016). This effect may be reinforced by classical and operant conditioning, even without any conscious awareness of the reinforcement ( Babel *et al.*, 2017; Becker *et al.*, 2008; Hölzl *et al.*, 2005; Klosterhalfen *et al.*, 2009) Classical conditioning may be associated with medical as well as non-medical cues ( Jensen *et al.*, 2015). For example, patients may feel nauseated after taking a strawberry-flavoured medicine if they experienced adverse effects after taking a medicine with that flavour in the past. Verbal suggestions and conditioning work synergistically, and that the magnitude of the effect is larger when it is caused by verbal suggestion and conditioning than by the verbal suggestion alone ( Petersen *et al.*, 2014). Negative information and conditioning cause negative expectations, negative attitudes towards treatment, and negative emotions ( Price, 2015) ( Benedetti *et al.*, 2003). The expectations, in the context of the nocebo effect, may be understood in the classical sense, as a set of negative beliefs regarding the effect of the treatment in general ( Elsenbruch *et al.*, 2012), but also as an assumption and immediate prediction of what will happen next ( Barsky & Borus, 1999; Bingel *et al.*, 2011) Conditioning may also happen without any explicit expectations ( Babel *et al.*, 2017; Bräscher *et al.*, 2017)

The nocebo effect may also be caused by a negative attitude to the treatment or dissatisfaction with either the treatment or the doctor. For example, patients often distrust generic drugs and believe that they are less effective and more harmful than branded drugs ( Al Ameri *et al.*, 2011; Himmel *et al.*, 2005). Patients who are switched to generic drugs tend not to adhere to treatment ( Labiner *et al.*, 2010) and report worse outcomes and more frequent adverse events ( Häuser *et al.*, 2012). Many doctors also think that generic drugs are of lower quality ( Heikkilä *et al.*, 2007), and unintentional cues that they give may make patients’ attitudes even more negative and enhance the nocebo effect ( Häuser *et al.*, 2012). Similarly, a negative consultation may evoke the nocebo effect. Patients expect a doctor to understand and recognise their problems (validation), give it a name (diagnosis), explain how it is going to progress (prognosis), and offer treatment, usually in a form of a medication. If any of the elements of the consultation is negative, for example, if a doctor dismisses patients’ complaints as being “all in their head”, patients may feel that their treatment needs were not met and that their sickness was invalidated ( Vangronsveld & Linton, 2012). This makes them feel hopeless and angry ( Häuser *et al.*, 2012) and increases the nocebo effect ( Barsky *et al.*, 2002). Such a negative effect of consultation may be stronger than the positive effects of consultation ( Greville-Harris & Dieppe, 2015) and may persist for a long time ( Blasini *et al.*, 2017); although clinicians’ positive suggestions may reduce the effect of these negative messages ( Crichton & Petrie, 2015).

## The nocebo effect in clinical trials

In clinical trials, the nocebo effect manifests as reduced improvement or increased frequency of adverse events, in both the placebo and treatment arms. Patients’ withdrawal from a trial due to these adverse events is also considered to be a nocebo effect ( Barsky *et al.*, 2002; Blasini *et al.*, 2017; Tobert & Newman, 2016). The nocebo effect in clinical trials is undesired and may distort the results of the trial; for example, if patients do not improve sufficiently, it may be concluded that the tested treatment is ineffective, or if patients report many adverse events, the conclusions may be that the treatment is harmful and the trial may be terminated early. Moreover, if these adverse events lead to the withdrawal of many participants, the missing data may further complicate interpretation of the results ( Mitsikostas *et al.*, 2011).

The nocebo effect is common, but, unlike the placebo effect, it is rarely discussed in the context of clinical trials, and it may not be considered when interpreting the results of a study. This phenomenon is under-recognised, and both the magnitude of the nocebo effect ( Petersen *et al.*, 2014) and the percentage of patients in clinical trials reporting adverse events as a result may be underestimated ( Amanzio *et al.*, 2009; Mitsikostas *et al.*, 2011; Rief *et al.*, 2006). For example, a meta-analysis of clinical trials of pharmacological treatments for neuropathic pain showed that about 52% (95% CI: 36-68) of placebo-treated patients reported adverse events and 6.0% (95% CI: 4.5-8.0) withdrew from a trial owing to adverse events ( Papadopoulos & Mitsikostas, 2012).

In clinical trials, as in the clinic, the nocebo effect may be introduced by negative information about the beneficial and adverse effects of the tested treatment that are described in the information letter or during the informed consent process, and not by the pharmacological properties of the treatment ( Barsky *et al.*, 2002). This may bias trial outcomes, especially if these outcomes are based on patients’ reports. For example, the frequency of reported gastrointestinal adverse events and discontinuation rates due to these adverse events in a trial of aspirin were much lower in a centre that did not include information about possible gastrointestinal bleeds than in two centres that included this information ( Cairns *et al.*, 1985; Myers *et al.*, 1987) ^.^ Moreover, about a quarter of patients taking placebo spontaneously report at least one adverse event, and this figure increases when they are actively asked about adverse effects ( Barsky *et al.*, 2002; Rosenzweig *et al.*, 1993).

Some symptoms may be wrongly attributed to the treatment, and this is more likely in patients with negative expectations ( Barsky *et al.*, 2002). Trial participants may focus their attention on new symptoms and interpret normal physiological sensations or benign symptoms, which may usually get little attention, as adverse effects of the treatment ( Barsky & Borus, 1999; Gurwitz *et al.*, 2003; Rosenzweig *et al.*, 1993). Such symptoms are typically generalised and nonspecific, for example, nausea, headaches, fatigue, or irritability. They are not associated with any disease and commonly occur in healthy people not taking any medications ( Eriksen & Ursin, 2004). For example, 77% of students responded that they had experienced at least one such symptom in the previous three days ( Reidenberg & Lowenthal, 1968). Moreover, some symptoms that are interpreted as adverse effects are highly prevalent in the populations for whom the drug is prescribed, for example headaches in women taking contraceptive pills ( Grimes & Schulz, 2011) or muscle problems in older patients taking statins ( Tobert & Newman, 2016). These “noise” symptoms may be misattributed to the treatment ( Barsky *et al.*, 2002; Grimes & Schulz, 2011; Tobert & Newman, 2016).

Not all nocebo-related negative effects events are “nonspecific” ( Rief *et al.*, 2009). Some complaints may be disease-specific, as patients may mistake symptoms of an underlying illness for adverse treatment effects ( Fine & Johnston, 1993). Many adverse events reported by patients taking placebo are typical of the treatment in the active arm ( Amanzio *et al.*, 2009; Barsky *et al.*, 2002; Blasini *et al.*, 2017; Rief *et al.*, 2009). For example, in a meta-analysis of trials of anti-migraine treatments, anorexia and problems with memory, which often occur in patients taking anti-epileptic drugs, were reported only in patients in the placebo arms of trials of anti-epileptic drugs ( Amanzio *et al.*, 2009). In another study, the rates of adverse events were much higher in the placebo arms of trials of tricyclic antidepressants than in trials of selective serotonin reuptake inhibitors, which reflects the adverse effects profiles of these classes of drugs ( Rief *et al.*, 2009). These examples demonstrate that information about adverse effects of different classes of drugs causes expectations that may influence the experience of adverse events and may bias clinical trial outcomes ( Rief *et al.*, 2009).

The trial design itself may introduce the nocebo effect and undermine its results. For example, random assignment to different treatment regimens means that patients are not given a choice, which may create a nocebo effect ( Bartley *et al.*, 2016). However, blinding of patients and assessors reduces placebo and nocebo bias, because it tends to make the conditions and expectations identical in the active and placebo groups ( Collins & MacMahon, 2007). Lack of blinding in one study arm (observational/waiting list group) or all study arms (open-label trials) may distort trial results, as knowledge about the received treatment may affect the incidence of reported adverse events. For example, in a group of patients who knew they were taking atenolol and that erectile dysfunction may a possible adverse effect the incidence of this symptom was 31%; in a group that was informed about the drug but not about the adverse effects, the incidence was 16%, and in the group that was blinded and not told explicitly about this potential effect the incidence was only 3.1%. In the patients who reported this adverse effect, sildenafil or placebo were equally effective in curing it ( Silvestri *et al.*, 2003).

Placebo control is useful not only to test whether the active treatment is more effective than placebo but also whether it is truly more harmful than placebo. Without a placebo control, all adverse events may be attributed to the active element of the treatment. For example, in a trial of statins, during the blinded and randomised phase, muscle-related symptoms were reported equally often in the active and placebo arms, but during the unblinded phase they were more frequent in patients taking statins ( Ganga *et al.*, 2014; Gupta *et al.*, 2017; Kashani *et al.*, 2006). Moreover, patients with well-documented statin intolerance due to muscle symptoms usually tolerate a statin under double-blind conditions ( Newman & Tobert, 2015). If patients in the control group do not receive placebo but are only followed-up in the trial (so-called observational/waiting list) they may demonstrate deteriorating symptoms or reduced improvement in self-limiting conditions, because they know they are left without any treatment and their treatment expectations are not met. For that reason, a non-interventional arm does not represent the natural history of the disease, because there is a double bias: not only are these patients not blinded, but their treatment expectations are not met, because they are left with no treatment, which leads to the nocebo effect and either worsening of their symptoms or slower recovery.

## Recommendations and future directions

Unlike improvement associated with placebo, there are no benefits related to the nocebo effect; it, therefore, has to be minimised by reducing pre-existing negative expectations or by preventing new ones ( Tobert & Newman, 2016).

Negative symptoms may not be reported if they are not prompted. It may be beneficial not to inform patients about potential adverse events that may be unrelated to the treatment or be of little clinical importance, such as mild headaches or nausea ( Tobert & Newman, 2016). However, it is crucial to warn patients about clinically important or potentially dangerous adverse effects predicted from the pharmacological properties of a drug; for example, warning patients not to drive or operate heavy machinery after medications that cause drowsiness. In clinical trials, it is also very important to record and include in the publication the exact content and phrasing of the information given to the participants, because it may have a substantial effect on the trial results. The nocebo effect can also be prevented by careful phrasing and positive framing of the information given to patients, for example by focusing on chances of improvement, survival, being symptom-free, and of not developing adverse effects ( Crichton & Petrie, 2015).

Another way to reduce the nocebo effect is to ask patients about their preconceptions and beliefs regarding a treatment or about previous experiences as they may negatively affect treatment outcomes. If patients’ beliefs are negative (if, for example, they think they are intolerant of the prescribed medicine), they will be more likely to report adverse events at follow-up ( Barsky *et al.*, 2002), especially when starting new medications ( Nestoriuc *et al.*, 2010). Such patients will also be less likely to adhere to treatment ( Barsky *et al.*, 2002), and may be more likely to stop taking the medication altogether ( Nestoriuc *et al.*, 2010). After a change of medication, patients with negative beliefs tend to report even more adverse events than during therapy with the original drug ( Nestoriuc *et al.*, 2010). It is therefore important to change patients’ attitudes before changing medications. Moreover, it may be worth asking patients to agree to a re-challenge with a drug they claim they do not tolerate ( Tobert & Newman, 2016), as having a choice is associated with better outcomes ( Botti & Iyengar, 2004). Finally, it is also important not to leave the patient without treatment, as any appropriately indicated treatment is better than staying on a waiting list ( Khan *et al.*, 2012).

## Conclusions

The nocebo effect is always negative and unwanted. It can easily be evoked by a careless word or unfortunate phrasing. It can also be learned from one’s own bad experiences or by observing others. It may also be caused by classical and operant conditioning, sometimes without patients being consciously aware of it.

Recognising the nocebo effect is important, because it may make a treatment look ineffective or harmful. For example, there may be no improvement or a much smaller improvement than expected, or the medication may seem to be poorly tolerated, with patients reporting many adverse events, which may lead to a change of therapy. However, patients who reported nonspecific complaints after one drug are likely to report even worse symptoms after a change of treatment.

The nocebo effect is also responsible for non-adherence to treatment and for discontinuation. When patients expect to feel worse or not to improve, they treat every negative sensation as being caused by the treatment, and so they do not take the treatment regularly or stop it altogether, which in turn results in a subtherapeutic dose of medication.

Any potential nocebo effect must, therefore, be recognised and minimised in the clinic and in clinical trials.

## Data availability

No data is associated with this article.
