# Supplementary material for: The nocebo effect as a source of bias in the assessment of treatment effects
Source: F1000Res. 2019 Mar 11;8:5. Originally published 2019 Jan 3. [Version 2] doi: 10.12688/f1000research.17611.2 (PMC6635979; doi:10.12688/f1000research.17611.2)
Supplement: Peer review history

# Peer review: Reviewer response for version 2

**DOI:** 10.5256/f1000research.20301.r50055

**Published:** 2019-07-15

**Authors:** Jian Kong

**Affiliations:** 1 Department of Psychiatry, Massachusetts General Hospital, Harvard Medical School, Charlestown, MA, USA

Avoiding the nocebo effect may have significant clinical implications by improving treatment effect and reducing potential side effects. In this manuscript, the author discusses basic and clinical studies on nocebo effect and concludes with recommendations on avoiding this negative effect. This is a well-written manuscript. My comments are as follows:

1. Not informing patients about negative treatment information, such as potential side effects or the possibility of no treatment response, may significantly reduce the nocebo effect. A critical issue here is how to strike a balance between telling the truth and not telling patients negative information. This can be very challenging as both law and ethics are involved. Thus, the author may also want to discuss the role of law, ethics, and culture in disclosing negative information that may produce the nocebo effect.
2. The author may want to include more brain imaging studies on nocebo and placebo effects, as well as the role of consciousness in these effects. Better understanding the brain mechanism underlying the nocebo effect and how this effect may be produced unconsciously may deepen our understanding of this important phenomenon.

I confirm that I have read this submission and believe that I have an appropriate level of expertise to confirm that it is of an acceptable scientific standard, however I have significant reservations, as outlined above.

## References

[1] : A functional magnetic resonance imaging study on the neural mechanisms of hyperalgesic nocebo effect. *J Neurosci*.2008;28(49) : 10.1523/JNEUROSCI.2944-08.2008 13354-62 10.1523/JNEUROSCI.2944-08.2008 19052227PMC2649754

[2] : A neural mechanism of direct and observational conditioning for placebo and nocebo responses. *NeuroImage*.2019;184: 10.1016/j.neuroimage.2018.10.020 954-963 10.1016/j.neuroimage.2018.10.020 30296557PMC6258205

# Response

**Published:** 2019-07-15

**Authors:** Karolina Wartolowska

**Affiliations:** University of Oxford, UK

I would like to thank the Reviewer for the excellent suggestions which I would address in my next manuscripts. The aim of this review was to give an overview of how the nocebo effect can arise and how it may distort the results of the treatment. I agree with the Reviewer that there have been many great neuroimaging studies investigating the neurobiological mechanisms underlying the nocebo. Several of these studies have been cited, including one by the Reviewer. The two publications, which have been suggested by the Reviewer and co-authored by the Reviewer, explore changes in activation and connectivity of pre-specified brain regions associated with pain processing and the nocebo response and may not be too specialised for researchers and clinicians who are not neuroscientists. I agree that the legal and cultural aspects of the nocebo effects associated with the information given by healthcare professionals are very important, but they are beyond the scope of this paper.

# Peer review: Reviewer response for version 2

**DOI:** 10.5256/f1000research.20301.r45544

**Published:** 2019-03-12

**Authors:** Przemysław Bąbel

**Affiliations:** 1 Pain Research Group, Institute of Psychology, Jagiellonian University, Kraków, Poland

The paper has been revised according to my comments. Although I still think that the role of classical conditioning and observational learning should be acknowledged in the recommendations and conclusions sections of the paper, I believe that the Author has reasons not to do it.

I confirm that I have read this submission and believe that I have an appropriate level of expertise to confirm that it is of an acceptable scientific standard.

# Peer review: Reviewer response for version 1

**DOI:** 10.5256/f1000research.19256.r42494

**Published:** 2019-02-04

**Authors:** Przemysław Bąbel

**Affiliations:** 1 Pain Research Group, Institute of Psychology, Jagiellonian University, Kraków, Poland

This is a well-written short summary of the studies on a very important issue which deserves much more attention than it actually gets. I have only two major concerns. First, the paper is focused mainly on verbal information as a source of the nocebo. Although I do agree that it is the most common source of the nocebo effect in clinical practice and clinical trials, two other sources are also important and their role should be discussed, i.e. previous experience (classical conditioning) and observation of other patients/participants of clinical trials (see for example 1). Especially, the role of those two additional sources should be included in the recommendations and conclusions sections of the paper.

 Second, through the paper the nocebo effect is discussed mainly in terms of negative expectations, however, it is only one of the explanatory mechanisms of the nocebo effect. Although nocebo effects induced by verbal information and observational learning are usually mediated by expectations, there is growing evidence that the nocebo effect induced by classical conditioning may not always be mediated by expectations (see references 2-8). Thus, I would rather avoid discussing the nocebo effect as the result of sole negative expectancies as well as I would avoid defining it in terms of negative expectations.

I confirm that I have read this submission and believe that I have an appropriate level of expertise to confirm that it is of an acceptable scientific standard, however I have significant reservations, as outlined above.

## References

[1] : Responding to nocebos through observation: social contagion of negative emotions. *Pain*.2013;154(8) : 10.1016/j.pain.2013.05.012 1165 10.1016/j.pain.2013.05.012 23707291

[2] : Differential Classical Conditioning of the Nocebo Effect: Increasing Heat-Pain Perception without Verbal Suggestions. *Front Psychol*.2017;8: 10.3389/fpsyg.2017.02163 2163 10.3389/fpsyg.2017.02163 29321752PMC5733554

[3] : Classical conditioning without verbal suggestions elicits placebo analgesia and nocebo hyperalgesia. *PLoS One*.2017;12(7) : 10.1371/journal.pone.0181856 e0181856 10.1371/journal.pone.0181856 28750001PMC5531508

[4] : In the face of pain: The choice of visual cues in pain conditioning matters. *Eur J Pain*.21(7) : 10.1002/ejp.1024 1243-1251 10.1002/ejp.1024 28497895PMC5509505

[5] : Not seeing or feeling is still believing: conscious and non-conscious pain modulation after direct and observational learning. *Sci Rep*.2015;5: 10.1038/srep16809 16809 10.1038/srep16809 26578164PMC4649469

[6] : A Neural Mechanism for Nonconscious Activation of Conditioned Placebo and Nocebo Responses. *Cereb Cortex*.2015;25(10) : 10.1093/cercor/bhu275 3903-10 10.1093/cercor/bhu275 25452576PMC4585522

[7] : Nonconscious activation of placebo and nocebo pain responses. *Proc Natl Acad Sci U S A*.2012;109(39) : 10.1073/pnas.1202056109 15959-64 10.1073/pnas.1202056109 23019380PMC3465419

[8] : Classical conditioning of analgesic and hyperalgesic pain responses without conscious awareness. *Proc Natl Acad Sci U S A*.2015;112(25) : 10.1073/pnas.1504567112 7863-7 10.1073/pnas.1504567112 25979940PMC4485119

# Response

**Published:** 2019-03-07

**Authors:** Karolina Wartolowska

**Affiliations:** University of Oxford, UK

I would like to thank the Reviewer for a very constructive review.  I agree with the Reviewer that the nocebo effect should not be defined only in terms of negative expectations regarding a treatment, especially, if the expectations are defined, in the narrow sense, as a set of beliefs about the treatment. However, the literature on nocebo uses the term “expectations” in a broader sense, being the negative state (conscious or subconscious) accompanying a treatment/therapeutic intervention. It comprises negative beliefs about treatment efficacy, negative emotions such as stress and anxiety, and anticipation and expectation of failure, lack of improvement or adverse effects. This state may be caused by previous bad experiences (either as a failed treatment or experimental classical conditioning), knowledge gained through experiences or information about treatment obtained from doctors, drug leaflets, media, other patients, by observing other patients or by learning from family and peers. As suggested by the Reviewer, the importance of classical conditioning and learning by observing others have been highlighted in the revised version of the manuscript.

# Peer review: Reviewer response for version 1

**DOI:** 10.5256/f1000research.19256.r43476

**Published:** 2019-01-30

**Authors:** Paul Dieppe

**Affiliations:** 1 University of Exeter, Exeter, UK

This is a useful review of a subject that, as the author says, deserves more attention than it gets.

 One of my concerns is that the abstract and opening sentences state that nocebo is a result of negative expectation, and yet, as mentioned later this is only one of a number of theories as to how the nocebo effect may be activated. Others include invalidation (mentioned briefly), conditioning (mentioned briefly) and activation of the fight or flight response (not mentioned at all).

 Another issue for me is the contexts in which nocebo can be an issue. This article mentions clinical trials, experimental settings such as pain perception, and clinical practice. I would find it easier to navigate the article if there was a clearer differentiation between these very different contexts, perhaps with subheadings.

 In relation to clinical practice, the article does not mention the fact that a consultation can make a patient’s disease or symptoms worse as a result of nocebo mechanisms.

 In the context of clinical trials I think that more attention should be given to the fact that the consent procedure can make symptoms worse, as well as resulting in a reduced response or adverse events.

 I found the English a little clumsy in places.

I confirm that I have read this submission and believe that I have an appropriate level of expertise to confirm that it is of an acceptable scientific standard, however I have significant reservations, as outlined above.

# Response

**Published:** 2019-03-07

**Authors:** Karolina Wartolowska

**Affiliations:** University of Oxford, UK

I would like to thank the Reviewer for their useful comments. I fully agree that expectations are only one of the mechanisms responsible for nocebo effect, but they are the one that is mentioned most frequently in the literature. Other mechanisms that may cause the nocebo effect, including stress or the “fight or flight” response, have also been discussed in this manuscript.

 I am grateful to the Reviewer for their comment on the lack of clarity regarding the subdivision of nocebo effect in clinical and trial contexts. In the revised version, additional subheadings have been added, and some paragraphs have been rearranged to follow the clinical/trial context subdivision followed by the possible causes and consequences of the nocebo effect. Hopefully, the new version of the manuscript is less clumsy and sufficiently highlights the role of conditioning, stress, consultation, and consent procedure in generating the nocebo effect.
